# Comment on: Chocolate Consumption and Risk of Coronary Heart Disease, Stroke, and Diabetes: A Meta-analysis of prospective Studies, *Nutrients* 2017, *9*, 688

**DOI:** 10.3390/nu9080852

**Published:** 2017-08-09

**Authors:** Gilberto F. Hurtado-Torres

**Affiliations:** Internal Medicine and Clinical Nutrition Department, Central Hospital Dr. Ignacio Morones Prieto and Faculty of Medicine, University of San Luis Potosi Mexico, V. Carranza Avenue 2395, San Luis Potosi SLP 78210, Mexico; gilberto.hurtado@uaslp.mx; Tel.: +444-834-2763

Shen Yuan et al. [1] highlight the protective cardiometabolic effects of chocolate intake. Without doubt, their work contributes to support evidence about the salutary and wide protective effects of chocolate compounds, particularly flavanols and its derivatives.

In the discussion, the authors state: “Our study is the first meta-analysis investigating the protective role of chocolate consumption against diabetes.”

However, this represents a slight imprecision; in 2011, Buitrago-Lopez et al. [2] published a meta-analysis where they explore the same final endpoints: cardioprotective and metabolic effect, diabetes mellitus included. They found a 31% diabetes mellitus reduction, underscoring that diabetes mellitus protective effect was derived just from one Japanese study published in 2010. The main difference between the studies by Buitrago-Lopez et al. and those of Shen Yang et al. is that the latter included in their meta-analysis four more studies which were published after the paper put forth by Buitrago-Lopez and colleagues. Thus, although the protective effect against diabetes mellitus was expressed as relative risk, both studies report close to the same range.

Another interesting difference between the two studies is that Shen Yang et al. found a chocolate protective effect occurred in the category of moderate chocolate consumption (<6 serving/week), while Buitrago-Lopez et al. found that a higher chocolate consumption (definitions of which vary from the number of servings per week or grams per day) confers the lowest incidence of diabetic and cardiometabolic events. Such differences deserve a detailed exploration in order to delimitate the ideal chocolate recommendation which provides optimal flavonoid amounts for a cardiometabolic protective effect [3] without increasing the risk derived from high caloric intake and eventual weight gain.
